# The Patient-Physician Relationship: Medical Students’ Perceptions in a Novel Course

**DOI:** 10.1007/s11606-024-08759-x

**Published:** 2024-04-10

**Authors:** Catherine Pressimone, Renusha Indralingam, Cameron Dowiak Metz, Arthur S. Levine

**Affiliations:** 1 University of Pittsburgh Medical Center, Pittsburgh, PA USA; 2 University of Pittsburgh School of Medicine, Pittsburgh, PA USA

## Abstract

The patient-physician relationship, especially in the case of severely ill patients, is often fraught with anxiety, grief, and guilt in the physician who may come to feel that he or she has failed the patient and thereby becomes a “second victim.” This notion was first explored in a 1973 publication (Artiss and Levine N Engl J Med 288(23):1210-4, 1973) that described a novel interactive seminar series for oncology fellows that had been designed to address and possibly remedy the frequent disquiet experienced by young physicians in this setting. Fifty years later, the medical student co-authors of this Perspective enrolled in an elective course that comprised a similar series of interactive seminars, now addressing the contemporary patient-physician relationship. The earlier paper was employed as a historical background, and the framework of the course then broadened such that the students considered the current environmental changes in medical practice (social, cultural, financial, legal, policy) that may be linked to the character of individual patient-physician relationships. This essay reports on the students’ perception of such relationships, and on the environmental elements that may be helpful or harmful to the well-being of both patients and physicians.

In 1973, *the New England Journal of Medicine* published a Special Article that described novel debriefing seminars that were initiated to help oncology fellows process the inappropriate anger, anxiety, and denial that they often experienced while working with terminally ill patients.^1^ In these seminars, the fellows openly discussed issues like physician and patient interdependency, the patient’s denial of illness, and physicians’ identification with their patients. Fortunately, the fellows reported significant relief after participating in these facilitated group seminars. Despite these seminars’ promising outcomes, plus the initiation of similar programs over the last 50 years,^2–4^ the patient-physician relationship often continues to be anxiogenic, compromising physicians’ wellness.^5,6^ Moreover, each new relationship holds the potential for a physician’s anxiety, but seminars such as these offer a mechanism for critically addressing and processing these anxieties when they emerge. Importantly, several more recent and substantial changes in the physician’s environment (social, cultural, financial, legal, policy) may be constructive, but some may further influence the patient-physician relationship.^7^ In response to our medical students’ often curious, if not troubled, perceptions of the contemporary and frequently complex relationship, Dr. Levine, co-author of the original 1973 article, established an elective course in 2022 to formally address current elements of physician well-being and to engage in an interactive forum discussing the patient-physician relationship from a student’s perspective. The style and construction of these seminars was similar to what was described earlier,^1^ with discussions focused on the students’ own experiences, readings from the literature, and insights shared between the students and their facilitator. This Perspective, co-authored by three participating students, summarizes these discussions. The framework of the course began with a history, i.e., the relationship between young physicians and their severely ill patients as described in the 1973 paper, and broadened into a consideration of the major, more recent influences on this relationship.

The students observed that contemporary trainees continue to struggle with anxiety after poor patient outcomes, primarily due to unrealistic expectations of physician stoicism following emotionally compelling events.^5,6^ In addition to formally debriefing this otherwise unvoiced anxiety, various strategies have been employed to mitigate the harmful consequences of physician guilt after patient mortality. Research has examined support groups, one-on-one counseling, and journaling.^2,4^ For example, teaching ICU trainees to have thoughtful conversations with patients and families may lessen the psychological burden after a poor patient outcome.^8^ However, despite attempts to acknowledge and address this phenomenon, grief, guilt, and self-doubt commonly burden physicians when they cannot save a patient^9,10^; the physician is the “second victim”.^11^ This victimhood compromises fruitful patient-physician relationships and is common among early trainees who are inexperienced with processing death.^12–14^ While most medical centers now offer resources to anxiety-ridden trainees, many studies show that physicians prefer intimate discussions with friends over employer-provided resources. It is possible that this preference originates from fear that expressing anxiety in the workplace could hinder professional advancement.^15,16^ While our students found the discussion of individual physician anxiety and compassion for one’s self to be compelling, had observed anxiety in residents and faculty, and had themselves experienced anxiety and the value of debriefing, they also explored self-doubt in an existential context.^17,18^

As noted earlier, the framework that had been established for this course required that the students explore the social, cultural, financial, and policy determinants that may affect the relationships of all physicians with their patients. Most of these issues are of recent vintage, but some may have existed in 1973 and had not been visible to physicians or their patients. Whichever the era, the physician burden is further exacerbated by restrictions on autonomy. A recent issue that greatly concerns this Perspective’s authors is how reductions in physician autonomy, particularly by governmental regulations, affect the patient-physician relationship. Some regulations are grounded in data despite demonstrating mixed efficacy in practice, such as the National Research Act and opioid prescription regulations.^19,20^ In extreme cases, moral bias influences legal regulations, leading to laws that contradict medical evidence and hinder appropriate clinical practice, such as the criminalization of marijuana and restrictions on elective abortion.^21–23^

The American healthcare system’s increasing complexity further limits physician autonomy. Restrictions from the Libby Zion case, the Health Care Quality Improvement Act, and the Ethics in Patient Referrals Act focused on patient safety.^24,25^ Newer constraints arise from business-centered, restrictive approaches to medicine and payment. For example, although well-intentioned with expanded healthcare access, the Affordable Care Act instituted reimbursement structures based on performance metrics that physicians argue are “not reflective of health outcomes” and incentivize providers to circumvent costs by avoiding complicated or higher-risk patients who may inevitably score poorly on metrics like hospital readmission rates.^24,26^

Simultaneously, patient trust in physicians has fallen substantially, even before the COVID-19 pandemic began. An “infodemic” has arisen with both accurate and false medical information easily available to patients.^27^ With declining health literacy, patients struggle to make informed decisions.^28^ The pandemic exacerbated the distrust, creating uncertainty about the rapid development of a COVID-19 vaccine and decreasing trust in science and medicine by proxy.^29^ Vaccines are now political rather than medical talking points, demonstrating how politicization and the “infodemic” are fueling patient distrust.

Despite these negative influences, many factors have improved the patient-physician relationship. Since 1980, substantial increases in racial and gender diversity among US medical school matriculants and faculty have benefited physicians and patients alike.^30–32^ Students from diverse classes report that they learn more from others compared to students from more homogenous classes. Physicians identifying as racial minorities are more likely to practice primary care in underserved areas, providing important bridges to local populations. Most importantly, patients paired with race-concordant physicians report increased trust, likeability, and intention to revisit that provider.^33^

In addition to diversity, the clinical care team is becoming more multidisciplinary, welcoming advanced practice providers (APPs) like nurse practitioners and physician assistants. Tasks historically completed by physicians are shifting to other team members, with APPs commonly treating more socially complex patients and physicians treating more medically complex patients.^34^ Research shows that patients are more willing to accept APPs for follow-up and preventive care while they prefer physicians for perceived “medical” tasks.^35^ However, the literature is scarce regarding how the expansion of APPs affects the patient-physician relationship.

Another improvement in patient-physician relationships is the codification of cultural and social determinants of health as central values of practice. The Centers for Disease Control and Prevention and World Health Organization specify social determinants as one of five major factors affecting health, enabling healthcare to shift from a historically paternalistic approach to a patient-centered approach.^36,37^ Contemporary physicians integrate patients’ social, cultural, and individual health beliefs into evidence-based treatment protocols, dramatically changing the patient-physician relationship. Some common changes found throughout the literature^38–40^ and observed in the authors’ personal experiences include:

- Patients defining the goals of their care
- Physicians acting as consultants who prioritize patient views via collaborative decision-making
- Patients having autonomy via mandated requirements of informed consent
- Physicians tailoring their communication and treatments to their patients’ cultural, social, and economic backgrounds

Trainee education now reinforces the prioritization of cultural values. Culturally informed care is being integrated into graduate medical education, continuing medical education, and undergraduate teaching through community outreach programs, written practice cases, and informal role modeling on inpatient rounds.^41–43^ Many schools teach students about culturally competent care of local indigenous populations.^44,45^ Overall, student education has increasingly focused on more patient-centered communication. Patients drive goal identification rather than abject acceptance, the physician is more of a consultant, and language is colloquial rather than technical. Journals abide by “inclusivity guidelines,” and the 21st Century Cures Act requires patients to have unobstructed access to their medical records.^46^

The patient-physician relationship has maintained many characteristics as described in 1973, but important new characteristics have emerged—some fruitful and some not. Three co-authors of this Perspective are female medical students, which is a huge shift from the gender homogeneity of physicians 50 years ago. However, most physicians continue to struggle with anxiety, self-doubt, and guilt in the face of unavoidable poor patient outcomes. Fortunately, modern physicians experience less professional stigma when they and their institutions acknowledge this anxiety. The increased patient-centeredness and focus on social determinants of health have strengthened the patient-physician relationship, but we contend with increasing public distrust in physicians and rising challenges to physician autonomy. It is a fragile balance.

Without question, the individual patient-physician relation and the environmental issues (social, cultural, financial, legal, policy) that affect all physicians are closely linked, and it is this link which is likely to challenge us most frequently. The authors encourage physicians, their trainees, and their institutions to examine these several issues proactively rather than contemporaneously: attempting to deal with a physician’s anxiety during or immediately after a time of crisis may magnify rather than prevent the anxiety, further eroding our patients’ trust in us. We conclude that interrogating the characteristics and complexities of the patient-physician relationship individually and collectively, on a continuing basis, should increase the existence of fruitful patient-physician interactions.
